# A case of anti-N-methyl-D-aspartate receptor antibody encephalitis associated with immature ovarian teratoma

**DOI:** 10.20407/fmj.2025-045

**Published:** 2026-05-14

**Authors:** Akiko Ohwaki, Kurumi Isomura, Kyohei Takada, Mayuko Ito, Ryoko Ichikawa, Yusuke Shimizu, Haruki Nishizawa

**Affiliations:** Department of Obstetrics and Gynecology, School of Medicine, Fujita Health University, Toyoake, Aichi, Japan

**Keywords:** Anti-NMDA receptor encephalitis, Immature ovarian teratoma, Impaired consciousness

## Abstract

**Introduction:**

Anti-N-methyl-D-aspartate (NMDA) receptor encephalitis is typically associated with mature cystic teratomas. We report a case of anti-NMDA receptor encephalitis associated with an immature ovarian teratoma, which is a malignant tumor.

**Case:**

A 16-year-old girl who presented with impaired consciousness and abnormal behavior was referred to our hospital. After evaluation upon admission, she was diagnosed with anti-NMDA receptor encephalitis associated with an ovarian mature cystic teratoma, and laparoscopic right salpingo-oophorectomy was performed. The postoperative pathological diagnosis confirmed the presence of an immature ovarian teratoma. The patient subsequently underwent immunoglobulin therapy, steroid pulse therapy, and plasma exchange for the treatment of encephalitis. Bleomycin, etoposide, and cisplatin chemotherapy was administered after her symptoms improved.

**Conclusions:**

In this case, early collaboration among the departments of neurology, obstetrics and gynecology, and emergency medicine enabled a prompt diagnosis and effective treatment. The patient’s symptoms were successfully ameliorated through early removal of the tumor and pharmacotherapy. Although anti-NMDA receptor encephalitis generally has a favorable prognosis, early diagnosis and initiation of treatment considerably affect the outcome. Clinicians should suspect anti-NMDA receptor encephalitis when a young patient presents with impaired consciousness or abnormal behavior and consider the possibility of underlying malignant tumors.

## Introduction

Anti-N-methyl-D-aspartate (NMDA) receptor encephalitis, which is a type of paraneoplastic limbic encephalitis, predominantly affects young women and is frequently associated with ovarian tumors.^1^ Although the overall survival prognosis of this disease is relatively favorable, it presents with a wide range of symptoms, such as convulsive status epilepticus and prolonged impairment of consciousness. In severe cases of anti-NMDA receptor encephalitis, respiratory management may be required. Therefore, prompt initiation of treatment is critical.^2^

We report a case of anti-NMDA receptor encephalitis associated with an immature ovarian teratoma, which is a rare malignant tumor.

## Case

### History of present illness

The patient was a 16-year-old girl (gravida 0, para 0) who presented to a local clinic with a 1-week history of anxiety. She was diagnosed with adjustment disorder and started anti-anxiety medication, but her symptoms did not improve. She subsequently developed abnormal behavior, including agitation and the utterance of unusual sounds. On the basis of progression of her symptoms, she was urgently referred to our hospital via a local emergency clinic for suspected encephalitis. On arrival, her Glasgow Coma Scale score was E4V4M6. Clinical findings included mild facial palsy, resting tremor, polyphrasia, and a low-grade fever (body temperature: 37.8°C). Laboratory tests showed the following elevated tumor markers: AFP concentration, 106.8 ng/mL; CA19-9 concentration, 151.3 U/mL; and CA125 concentration, 12.7 U/mL. Routine blood tests were unremarkable. A cerebrospinal fluid analysis showed clear fluid with no xanthochromia, the glucose concentration was 56 mg/dL, the total protein concentration was 23 mg/dL, the cell count was 8/μL, and no virus was detected.

**Figure 1:**
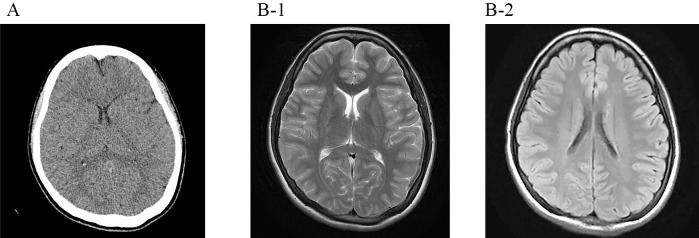
Head imaging findings. A: Plain head CT scan (axial section). B: Plain head MRI axial section (B-1: T2 weighted imaging B-2: fluid-attenuated inversion recovery pattern). No obvious abnormalities were observed on head imaging on the day of symptom onset.

**Figure 2:**
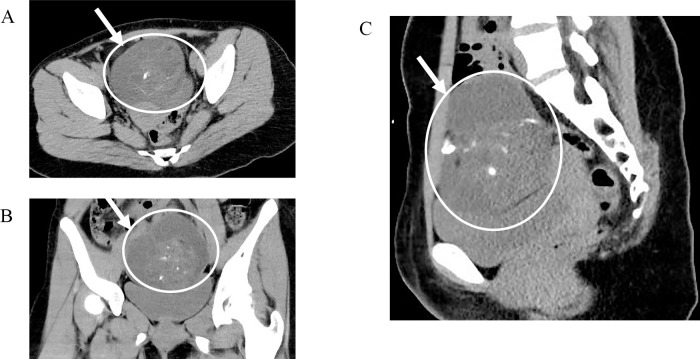
Abdominal plain CT scan. A: Cross-section, B: coronal section, and C: sagittal section. CT shows an intrapelvic tumor with internal calcification and fluid content containing fat (arrow).

Head computed tomography (CT) and magnetic resonance imaging (MRI) showed no abnormalities (Figure 1). However, contrast-enhanced abdominal CT showed a pelvic mass that measured 87×63 mm with internal low-density areas suggestive of fat and calcification (Figure 2).

### Post-hospitalization clinical course

The patient was suspected to have anti-NMDA receptor encephalitis and was referred to our department after whole-body screening showed a pelvic mass suspected to be an ovarian teratoma. Based on the patient’s young age, absence of psychiatric history, and the presence of prominent psychiatric symptoms, such as unusual vocalizations and impaired consciousness, her clinical presentation was consistent with probable anti-NMDA receptor encephalitis.^1^ A definitive diagnosis by cerebrospinal fluid testing was expected to take several days, and early intervention is essential in this condition. Therefore, we decided that surgical resection of the suspected tumor should be performed and scheduled the procedure for the day of admission.

### Surgical findings

**Figure 3:**
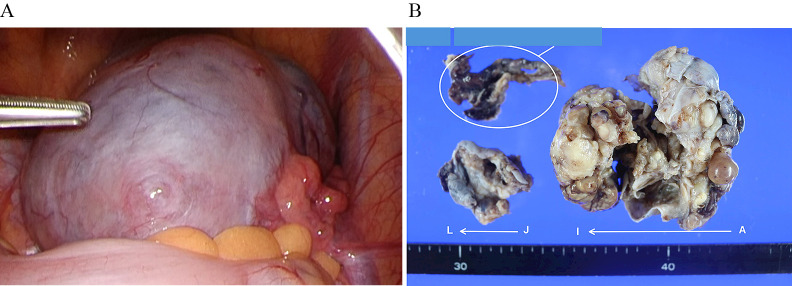
Tumor findings. A: Intra-abdominal images. B: Gross tumor findings. The right ovary was swollen to the size of a fist. Sebum and hair components were observed within the resected tumor.

The patient underwent laparoscopic right salpingo-oophorectomy. Intraoperatively, the right ovary was enlarged to the size of a fist, without adhesion to surrounding tissues. The resected tumor contained sebum and hair components. The procedure lasted 1 h and 26 min, with minimal blood loss and no intraoperative complications (Figure 3).

### Histopathological examination

**Figure 4:**
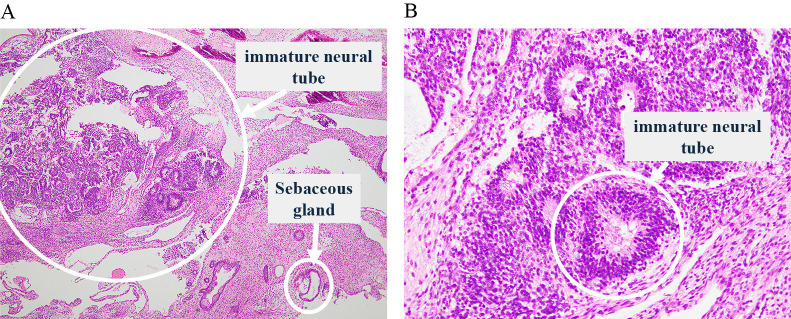
Pathological findings. A: Hematoxylin and eosin staining, 4×. B: Hematoxylin and eosin staining, 400×. Cysts lined with squamous epithelial tissue were observed and they contained various tissues, such as sebaceous glands, glandular epithelium, adipose tissue, smooth muscle, cartilage, bone, central nervous tissue, and salivary gland-type secretory glands (arrow). In some central nervous tissues, tubular aggregation of cells was observed. More than one immature neural tube component (arrow) was identified per field of view at 4× objective magnification, corresponding to Grade 2.

A multilocular cystic lesion lined with squamous epithelium was identified. Internally, the cysts contained keratinized material, calcification, cartilage, and fatty components (Figure 4). Tissues, such as sebaceous glands, adipose, smooth muscle, cartilage, bone, central nerves, and salivary gland-type secretory glands, were observed. In some central nerve tissues, there was an area in which cells were aggregated tubularly, and the Ki-67 labeling index was high. Immature neural tube components were observed with at least one per field of view at an objective magnification of 4× and were equivalent to grade 2. The central nervous tissue also showed marked lymphocytic infiltration.

### Postoperative course

**Figure 5:**
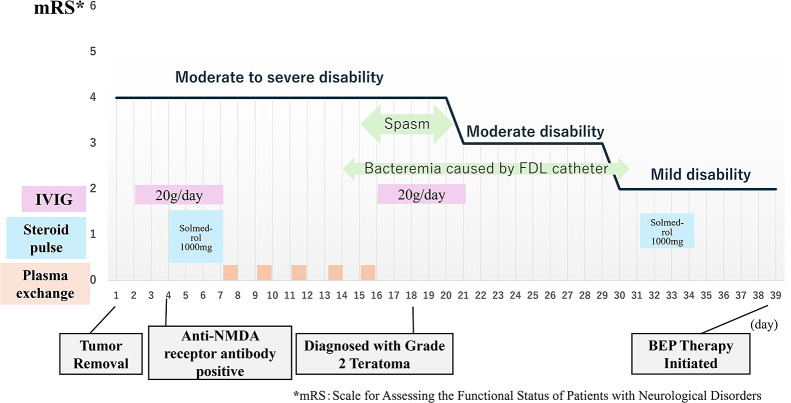
Clinical course of the patient.

**Figure 6:**
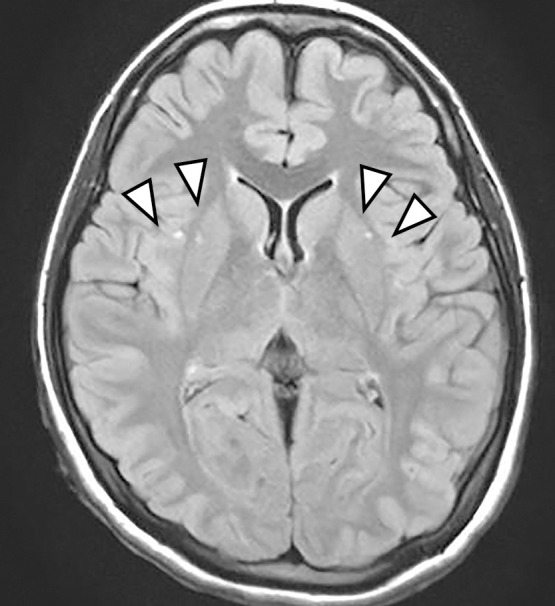
Post-treatment head MRI axial section with fluid-attenuated inversion recovery. High-intensity areas appeared in the bilateral lateral putamina and mild high-intensity areas were observed in the hippocampal limbic system (arrows), which are consistent with findings of anti-NMDA receptor encephalitis.

A preoperatively collected cerebrospinal fluid sample obtained on day 4 of illness was analyzed at BML Inc. (Tokyo, Japan). A positive anti-NMDA receptor antibody result confirmed the diagnosis (Figure 5). A postoperative MRI of the head showed small high-intensity areas in the bilateral lateral putamina and mild high-intensity areas in the hippocampal limbic system on fluid-attenuated inversion recovery imaging. These findings were consistent with anti-NMDA receptor encephalitis (Figure 6).^3^

Postoperatively, we initiated combination therapy with intravenous immunoglobulin (20 g/day) for 5 days and steroid pulse therapy (1000 mg/day) for the treatment of encephalitis after confirming that the inflammatory response was not considerably elevated. This pharmacotherapy, along with surgical antigen removal, led to a gradual improvement in the symptoms of encephalitis. Additionally, plasma exchange therapy for antibody removal and a second course of immunotherapy were administered, which resulted in the resolution of symptoms. The treatment for encephalitis was completed on day 31 of illness.

The histopathological diagnosis of the resected ovarian tumor, which was obtained on day 18 of illness, confirmed an immature teratoma. The symptoms of encephalitis improved and the patient regained the ability to understand her condition. Therefore, she was transferred to the Department of Obstetrics and Gynecology for bleomycin, etoposide, and cisplatin therapy to target the immature teratoma on day 38 of illness, following the completion of encephalitis treatment.

## Discussion

Anti-NMDA receptor encephalitis was first described in 2007 by Dalmau et al.^1^ This disease is a type of paraneoplastic limbic encephalitis characterized by the production of autoantibodies in the serum and cerebrospinal fluid against the NMDA receptor, which is a subtype of glutamate receptor. Anti-NMDA receptor encephalitis is believed to result from extensive immune-mediated damage to the central nervous system, particularly the limbic system, because of antibodies targeting surface antigens expressed in neural tissue within tumor cells. Key features of anti-NMDA receptor encephalitis include the presence of anti-NMDA receptor antibodies in cerebrospinal fluid, a higher incidence in young women, and a strong association with tumors, particularly ovarian teratomas. Clinical manifestations of this disease often begin with psychiatric symptoms resembling schizophrenia and may progress to include fever, seizures, and involuntary movements. Additionally, in severe cases, central hypoventilation can occur. These complications can necessitate ventilator support or result in prolonged unconsciousness. Despite the severity, this condition generally has a favorable prognosis when appropriately treated. Other features of this disease include the absence of characteristic findings on head CT or MRI, as well as nonspecific cerebrospinal fluid findings. The diagnosis of anti-NMDA receptor encephalitis is based on the established criteria for probable or definite anti-NMDA receptor encephalitis.^4^ The identification of immunoglobulin G anti-GluN1 antibodies is essential for a definitive diagnosis, and using an appropriate detection method with cerebrospinal fluid rather than serum is important (Table 1). Treatment strategies for encephalitis are determined on the basis of whether this disease is tumor-associated and the patient’s response to immunotherapy. When a tumor is present, the combination of tumor resection and immunotherapy is considered standard care.

In Japan, anti-NMDA receptor encephalitis was first described in 1997 by Nishimura et al.^5^ as a type of encephalitis that presents with psychiatric symptoms in young women and follows a prolonged course after severe acute-phase symptoms, such as impaired consciousness and convulsions. Despite the acute severity of anti-NMDA receptor encephalitis, it typically has a favorable long-term prognosis. In 2004, Kamei et al.^6^ further characterized this condition as acute juvenile female non-herpetic encephalitis, which also primarily affects young women. Following the report by Dalmau et al.^1^ on anti-NMDA receptor encephalitis, Iizuka et al. showed the presence of anti-NMDA receptor antibodies in serum and cerebrospinal fluid samples from four patients who were clinically diagnosed with acute juvenile female non-herpetic encephalitis and identified ovarian teratomas in three of the four patients. As a result, acute juvenile female non-herpetic encephalitis is currently considered essentially the same disease as anti-NMDA receptor encephalitis.^7^

Regarding the frequency of concomitant tumors, approximately 90% of cases of anti-NMDA receptor encephalitis are associated with ovarian teratomas, including benign mature cystic teratomas (67%) and malignant immature teratomas (25%), as reported by Dalmau et al.^1^ and Titulaer et al.^2^ Although there have been no direct comparisons of different ovarian tumor subtypes regarding their association with encephalitis, malignant tumors have been reported to be more frequently associated with anti-NMDA receptor encephalitis (approximately 14%) compared with sporadic teratomas (<3%).^8–14^ The pathogenesis of this disease is believed to be initiated by infiltration of teratoma tissue by inflammatory cells. Specifically, the immune system may lose tolerance when these inflammatory cells infiltrate intratumoral neural tissue, leading to the production of antibodies against NMDA receptors. The fact that teratomas associated with anti-NMDA receptor encephalitis tend to contain abundant neural tissue is considered pathogenetically significant. Previous studies showed that neural elements were present in all but one case of ovarian teratomas associated with anti-NMDA receptor encephalitis, whereas such elements were found in only approximately 30% of mature ovarian teratomas not associated with encephalitis.^7,15^ Therefore, the presence of intratumoral neural tissue appears essential in breaking immune tolerance and triggering the development of anti-NMDA receptor encephalitis. Regarding malignant tumors, additional treatment should be considered on the basis of progression of encephalitis because a variety of therapeutic approaches and outcomes have been reported in previous studies on immature teratomas. These studies included a case in which symptoms improved only after chemotherapy, following unsuccessful surgery and immunotherapy,^16^ and a case in which symptoms improved with surgery and immunotherapy alone.^17^ In another study, the patient was managed conservatively without early chemotherapy according to clinical assessments using the modified Rankin Scale and Performance Status scale.^18^ Overall, favorable treatment responses have been observed in patients with tumor-associated encephalitis.^12,19^ In cases of anti-NMDA receptor encephalitis where tumors were surgically removed, a favorable treatment responsiveness and prognosis have been reported. Improvements include symptomatic relief within 4 weeks postoperatively and enhanced scores on the modified Rankin Scale, which is a measure of neurological disability, within 24 months.^2^ Furthermore, one study showed considerable improvement in neurological symptoms and the overall prognosis when tumors were resected within 4 months of symptom onset.^10^ In contrast, another study reported a higher incidence of recurrence, a more prolonged disease course, and a poorer prognosis in patients whose tumors were not removed.^2^

As mentioned above, in anti-NMDA receptor antibody encephalitis complicated by malignant tumors, evaluation of tumor characteristics before initiating encephalitis treatment for encephalitis is important for subsequent management. Although imaging may suggest an immature teratoma based on calcification patterns, a definitive diagnosis is difficult, and the requirement for urgent surgery often limits opportunities for intraoperative rapid assessment. Moreover, no studies have examined outcomes according to the surgical procedure for ovarian tumors in this context. Despite variability among cases and institutions, unilateral adnexal resection remains a reasonable option, particularly when fertility preservation is desired, because it may reduce surgical invasiveness and the risk of tumor content spillage. Therefore, while a preoperative and intraoperative rapid diagnosis can assist in planning postoperative management, it does not affect decisions regarding the treatment of encephalitis and may thus be considered optional.^20,21^

In the present case, anti-NMDA receptor encephalitis was suspected early, and the patient underwent whole-body imaging shortly after admission. This approach allowed for prompt initiation of treatment in collaboration with the departments of neurology, gynecology, and anesthesiology. The clinical course was considered favorable because the patient’s condition improved within 1 month following tumor resection.

## Conclusions

We report a case of anti-NMDA receptor encephalitis associated with an immature ovarian teratoma. This disease, characterized by impaired consciousness and psychiatric symptoms in young women, requires a multidisciplinary and timely approach. When an ovarian tumor is identified, prompt surgical resection combined with immunotherapy is essential for improving outcomes. Additionally, careful selection of surgical procedures and comprehensive management are necessary, especially considering the potential for malignant tumors.

## Figures and Tables

**Table 1:** Diagnostic criteria for probable anti-NMDA receptor encephalitis

Probable	
1. At least four of the following six primary symptoms rapidly appear within three months.	
a.	Abnormal mental or behavioral abnormalities, or cognitive impairment
b.	Language disorders (verbosity, reduced speech, mutism, etc.)
c.	convulsion
d.	Abnormal movements (dyskinesia, rigidity, postural abnormalities, etc.)
e.	Decreased level of consciousness
f.	Dysfunction or Central Hypoventilation
2. At least one positive test result is observed.	
a.	Abnormal EEG findings (focal or diffuse slow wave activity, disruption of the background rhythm, epileptic activity, or extreme delta brush)
b.	Increased cerebrospinal fluid cells or positive oligoclonal IgG bands
Other diseases can be excluded.	
However, in cases with teratomas, a diagnosis of probable anti-NMDA receptor antibody encephalitis can be made if three of the above primary symptoms are present.	
Definite	
After excluding other diseases, the presence of one or more of the above six primary symptoms, and IgG-type anti-NMDAR antibody positivity (GluN1 antibody)	

*Diagnosis criteria for anti-NMDA receptor encephalitis* ^1^
